# A Brief Review on the Neuroprotective Mechanisms of Vitexin

**DOI:** 10.1155/2018/4785089

**Published:** 2018-12-05

**Authors:** Layana Karine F. Lima, Suyanne Kássia S. Pereira, Ronaldo dos Santos S. Junior, Felipe Pereira da S. Santos, Alyandra de Sousa Nascimento, Chistiane Mendes Feitosa, Juliana de Sousa Figuerêdo, Antonio do Nascimento Cavalcante, Edigênia Cavalcante da C. Araújo, Mahendra Rai

**Affiliations:** ^1^Post-Graduate Programs in Pharmaceutical Sciences, Federal University of Piaui, Ininga, 64.049-550, Teresina, Piauí, Brazil; ^2^Post-Graduate Programs in Chemistry, Federal University of Piaui, Ininga, 64.049-550, Teresina, Piauí, Brazil; ^3^Univasf, Collegiate of Pharmaceutical Sciences, Avenue José de Sá Maniçoba, s/no, Center, CEP 56304-205 Petrolina, PE, Brazil; ^4^Department of Biotechnology SGB Amravati University, Amravati PIN-444602, Maharashtra, India

## Abstract

The neural dysfunction is triggered by cellular and molecular events that provoke neurotoxicity and neural death. Currently, neurodegenerative diseases are increasingly common, and available treatments are focused on relieving symptoms. Based on the above, in this review we describe the participation of vitexin in the main events involved in the neurotoxicity and cell death process, as well as the use of vitexin as a therapeutic approach to suppress or attenuate neurodegenerative progress. Vitexin contributes to increasing neuroprotective factors and pathways and counteract the targets that induce neurodegeneration, such as redox imbalance, neuroinflammation, abnormal protein aggregation, and reduction of cognitive and/or motor impairment. The results obtained provide substantial evidence to support the scientific exploration of vitexin in these pathologies, since their effects are still little explored for this direction.

## 1. Introduction

Neurodegenerative dementias and movement disorders are becoming more common, mainly as a result of increased life expectancy and changing demographic population [1]. According to estimates by the World Health Organization (WHO) more than 47 million people worldwide live with dementia and by 2030, and the number is being speculated to increase to 75 million [2] (WHO, 2017). The currently available treatments are able to improve the symptoms of the disease without delaying or disrupting its progression, so they cannot prevent the underlying neurodegenerative conditions.

Therefore, the search for drugs capable of inhibiting or delaying neural loss has mobilized research on natural products and their derivatives as safer and more accessible alternatives. In this context, the Vitexin (apigenin-8-C-glycosidic), a flavonoid found in several medicinal plant species such as* Ficus deltoida* [3],* Spirodela polyrhiza* [4], and* Acer palmatum* [5] has been the subject of studies due to its pharmacological activities, including antioxidant activity [6], anti-Alzheimer's [7], and neuroprotective activity [8, 9]. In addition, studies have shown that flavonoids, such as vitexin, can modulate gene transcription and phosphorylation of pathways of death and cell survival [10, 11], key events of degeneration neural. In this sense, vitexin, as flavonoid, may act on several therapeutic targets to prevent or reduce the events involved in neurodegeneration, as we will see below.

## 2. General Considerations about Neurodegeneration

Neurodegenerative diseases (ND) are multifactorial debilitating disorders characterized by neuronal dysfunction, with progressive loss of neurons in specific regions of the brain and spinal cord [12]. The main manifestations of this neuronal deficiency are progressive loss of memory, cognitive impairment, dysfunction locomotor, and emotional and behavioral problems observed in individuals with Alzheimer's, Parkinson's, Huntington's, and Amyotrophic Lateral Sclerosis [13, 14].

**Figure 1:**
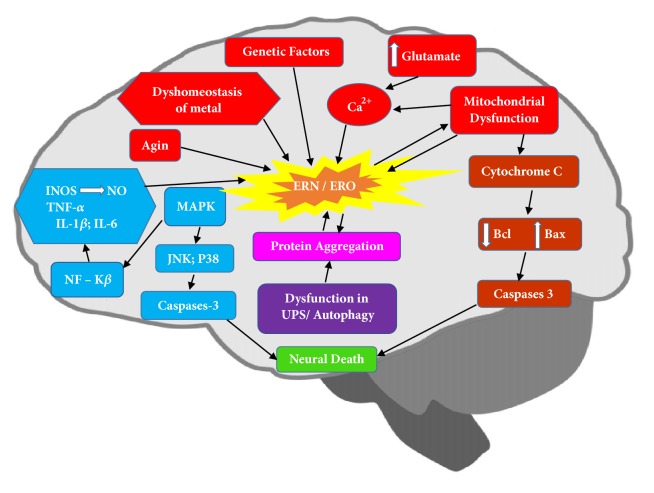
The neurotoxicity induced by the presence of reactive oxygen and nitrogen species and toxic oligomers occurs in a positive feedback process and is associated with an exacerbated inflammatory response, glutamate excitotoxicity, mitochondrial dysfunction, and abnormalities in the ubiquitin-autophagic proteasome system. These events promote the release of calcium and cytochrome c by mitochondria and activate the apoptotic signaling pathways that result in cell death and neurotoxicity. In addition to normal aging, factors such as genetic and environmental changes are the main triggers of neurodegeneration.

Beyond normal brain aging, events which cellular and molecular such as increased stress oxidative [15], neuroinflammation [16], impairment of function mitochondrial [17], activation of factors apoptotic [18], signaling cellular altered [19], altered autophagy, chronically activated microglia, and gene expression are also involved in neural loss as shown in Figure 1 [20].

Neurodegenerative diseases and ischemia have main triggering factor stress oxidative (EO) [21]. The presence in the brain of a rich content of polyunsaturated fatty acids, high aerobic rate, presence of transition metals, and reduced antioxidant enzymatic activity are factors that make it susceptible to oxidative damages [15]. With the aging and decreased ability of cells to maintain redox balance, there is accumulation of free radicals, mitochondrial dysfunction, and neuronal damage that together lead to the initial and progressive linkage of neuromogenic diseases [22, 23].

Level increase of Oxigen Reactive Species (ORS) and Nitrogen Reactive Species (NRS) stimulates the release of proinflammatory cytokines, chemokines, and induce neuroinflammation [24]. Although inflammation is an endogenous defense mechanism, its prolonged state promotes the progression of neurodegenerative diseases [25]. In neuroinflammation the activation of primary immune cells in the brain, astrocytes, and microglia occur by stimulating transcription of proinflammatory genes [26]. The sustained state of activated microglia results in overproduction of proinflammatory mediators such as tumor necrosis factor *α* (TNF*α*), nuclear factor *κ*B (NF-*κ*B), and nitric oxide (NO) [27]. Through NOx reactions, NO forms the neurotoxic peroxynitrite radical (ONOO-), responsible for the oxidation of biological molecules, besides promoting the nitration of proteins [28]. The oxidation and nitration of proteins result in alteration of the function of many enzymes involved in the transport of mitochondrial electrons and triggers the release of calcium from the cytochrome c and ORS mitochondria resulting in a vicious cycle with consequent apoptosis [29].

Oxidative stress (OS), the influx of Ca^2+^, and proinflammatory cytokines, such as tumor necrosis factor (TNF) –*α* and interleukin (IL) -1*β*, activate apoptotic signaling pathways mediated by the mitogen-activated protein kinases (MAPKs) JNK and p38 and induce the gene expression of *β*-secretase [4]. MAPK is also activated by *α*-synuclein aggregates, which in addition to forming Lewy bodies form oligomers that activate microglia and stimulate the expression of inflammatory mediators and of ORS with consequent release of cytochrome c, by mitochondria, and death of [30].

Another factor that triggers neurodegeneration is the reduction of the clearance of deformed, damaged, aggregated, or unnecessary proteins with consequent accumulation of protein aggregates [31]. Protein catabolism deficiencies are present in many neurodegenerative diseases and are associated with a deregulation of the ubiquitin proteasome system (UPS) and the autophagia-liposomal system [32].

## 3. Neuroprotective Effects of Vitexin

### 3.1. Alzheimer's

Abnormal aggregation of A*β* peptides is a pathological marker of Alzheimer's disease (AD) and has a direct relationship with exacerbated oxidative stress and mitochondrial dysfunction [33]. In this line of reasoning, research is aimed at investigating the protective effect of vitexin against this aggregation and reduction of its toxicity begins to emerge.

In Alzheimer's disease the oxidative stress plays an important role in the onset and progression of pathology [34]. The direct relationship between OS and the accumulation and abnormal aggregation of specific forms of A*β* peptides in senile plaques occurs through a positive feedback mechanism, where the generation of A*β* contributes to the generation of OS and this in turn activates the polymerization of the beta-amyloids peptides [35]. In a study by Malar et. al. [36], Vitexin (50 *μ*M) was able to reduce the formation of ROS and NRS and the markers of protein and lipid oxidation, carbonyl protein, and malondialdehyde and to reduce loss of membrane potential by a mechanism of increased mitochondrial biogenesis; induction of the expression of antioxidant genes nucler factor 2 (Nrf-2) and heme oxygenase-1 (HO-1) with increased GSH level; inhibition of apoptotic signaling pathways activated by caspase-3 and bax; and increased expression of the antiapoptosis protein Bcl-2. In that same study vitexin (10 *μ*M) prevented the interaction between peptides and their aggregation, essential for the induction of toxicity in Neuro-2*ª*  cells by regulating cholesterol homeostasis, and indirectly contributed to its degradation by USP. Failures in protein catabolism are associated with a deregulation of the ubiquitin-proteasome system (UPS) and the autophagia-liposomal system [32]. In familial Alzheimer's disease, the dysfunction of presenilin 1, a catalytic component of *γ*-secretase, required in the formation of *β*-amyloid, compromises acidification of lysosomes and results in inhibition of proteolysis and accumulation of A*β* peptides in autophagosomes [37, 38]. The accumulation of A*β* peptides may also be related to the reduced presence of Beclin1, a positive regulator of autophagy, and contribute to inhibit proteasome activity [38].

The Vitexin has also been shown to limit the formation of beta-amyloid peptides by inhibiting the enzyme BACE1, a *β*-secretase that catalyzes the proteolysis of the amyloid precursor protein (APP) in Alzheimer's [39]. The protein Beta-amyloid is produced by sequential proteolytic cleavages of APP that is cleaved sequentially by two membrane-bound proteases, the beta-cleavage enzyme of the beta 1 site (BACE1) also called *α*-, *β*-secrettases and *γ*-secretases [39]. These aggregated proteins cause a proteasomal dysfunction and generate a vicious cycle of accumulation of oxidized proteins and subsequent aggregation of proteins and thus further stimulate the formation of reactive species and subsequent excitotoxicity when interacting with glutamate receptors [40].

The presence of glycosylation C-8 in flavonoid vitexin is related to reduced levels of apoptosis and NO induced by the A*β* peptide, in cells PC12 (rat adrenal pheochromocytoma cell line) when compared to untreated cells. This increase in viability was observed by reducing the levels of lactate dehydrogenase (LDH) in a dose-dependent manner, with a maximum increase in viability in vitexin treatment at 150 *μ*g/mL [41]. This organic characteristic of the molecule is also attributed to potent inhibition of cholinesterase types (AChE and BChE) in RAW 264 cells, but in this study vitexin did not inhibit the production of reactive NO species in LPS-stimulated cells, possibly by having them tested at very low concentrations (5, 15, and 30 *μ*M) [7]. These results show that both formation and beta-amyloid peptide induced toxicity, involved in the pathogenesis of AD, can be coated by vitexin at safe concentrations, thus contributing to the reduction of neural degeneration, whereas inhibition of enzymes AChE and BChE contributes to the improvement of cognition [42].

### 3.2. Parkinson

Due to the presence of enzymes generating of ORS, such as tyrosine hydroxylase and monoamine oxidase, dopaminergic neurons are particularly prone to oxidative stress [43]. The breakdown or degradation of dopamine by monoamine oxidase can produce 6-hydroxydopamine and hydrogen peroxide, which in the presence of high levels of iron is converted to hydroxyl radicals, toxic to neuronal cells [44, 45]. As in Alzheimer's disease mitochondrial complex I dysfunction can affect dopaminergic neurons and is associated with increased production of mitochondrial ORS and redox signaling [46].

In models of simulation of Parkinson disease (PD) in Vitexin (10 to 100 *μ*M) reduced cytotoxicity and poptosis in human neuroblastoma SH-SY5Y cells, induced by the active metabolite of 1-methyl-4-phenyl-1,2,3,6-tetrahydropyridine (MPTP). In addition, vitexin also contributed to increase the viability of these cells by positively regulating the phosphorylation of the PI3K/Akt survival signaling pathway and thereby reduce the high Bax/Bcl-2 ratio and the expression of caspase-3, both in vitro and in vivo, when compared to the reference drug in PD therapy, in mice it contributed to attenuate symptoms such as motor deficit and bradykinesia [47]. The active metabolite (MPP^+^) is a parkinsonian toxin that inhibits the mitochondrial complex I and generates oxygen free radicals, its effects produce symptoms similar to those observed in PD, such as bradykinesia and loss of neuronal dopaminergic cells SN [48], and the reversal of its effects by vitexin is crucial for a good prognosis of PD, since the involvement of apoptotic and antiapoptotic signaling pathways triggers undesirable phenotypes such as microglia activation, neuroinflammation, oxidative stress, and apoptosis [49].

Current therapies for patients with Alzheimer's and Parkinson's are focused on improving symptoms, such as increased acetylcholine in AD, with benefits in memory and cognition, and those reducing Parkinson's dopamine degradation without delaying its progression [50]. These therapies are palliative and do not operate in the pathological genesis of neurodegeneration, such as the reduction of OS and neuroinflammation, as well as the modulation of signaling pathways for death and survival of cells. Vitexin has been shown to act in these pathways in preclinical experiments with results that qualify it as a promising molecule for further investigations and development of an adjuvant drug for the treatment of those diseases.

### 3.3. Insults Acute Encephalic

Neurodegenerative diseases are associated with increased ORS/NRS and chronic inflammatory response and culminate with mitochondrial dysfunction, calcium dysregulation, glutamate hyperactivity and protein aggregation with consequent cognitive dysfunction, cellular damage, and apoptosis. These events are also present in acute insults such as ischemia, encephalic vascular accident and anesthetic induced neurotoxicity. Further,* in vitro* and* in vivo* studies show how vitexin may interfere with a better prognosis associated with various injuries in the Central Nervous System (CNS).

In rat brain, with diabetes induced by streptozotocin (STZ) [51], it was found that vitexin (1 mg/kg) conferred a neuroprotection by lowering TBAR levels, lipid peroxidation products, and improving the metabolism of glucose in the brain. They also observed that vitexin had an effect on the improvement of learning and memory observed in the Morris's water maze (MWM), the authors suggest that this effect of vitexin is associated with an increase in cortical glycation that in diabetic rats is decreased and increased antioxidant enzymes SOD and glutathione peroxidase (GPx).

When investigating the effects of vitexin on brains of young rats Min* et. al.,* [52] found that acute vitexin (30-60 mg/kg) decreased levels of hypoxia-inducible factor-1*α* (HIF-1*α*) and factor of vascular endothelial growth (VEGF), which were significantly increased after ischemic hypoxia in neonates, reduced lesions, stroke volume and cerebral edema. In addition, early inhibition of apoptosis inducing HIF-1*α* has preserved the blood-brain barrier of ruptures, attenuated neuroinflammation, brain tissue loss, and atrophy observed following hypoxia-ischemic injury in the developing brain, both in the early stages and after two weeks of vitexin treatment. The authors associated these findings with the improvement of sensorial and motor deficits caused by ischemia.

In accordance with the above study, sevoflurane anesthetic-induced neurotoxicity in vivo and in vitro was reduced by administration of vitexin at 50 mg/kg and 100 *μ*M, respectively, in the experiments performed by Luy et al., [53] and vitexin still reduced the content of MDA and increased levels of antioxidants SOD and GSH-px. Also in this study, the expression of HIF-1*α* and VEGF expression in both newborn and human (H4) cell cultures of the human neuroglioma (H4) cells was decreased, thus vitexin increased the susceptibility of these cells by negatively regulating late apoptosis by the p38 protein via the MAPK signaling pathway and as a consequence the expressions of the proapothetic proteins caspase-3 and Bax were inhibited.

This protective effect of vitexin on reducing the volume of cerebral infarction and preserving cortical and hippocampal cells was also evidenced in cerebral ischemia / reperfusion injury in a preclinical model of middle cerebral artery occlusion (MCAO) in mice. In this experiment, vitexin at low concentrations (3.25, 7.50, and 15.00 mg/kg) showed neuroprotection by increasing the phosphorylation of the ERK1/2 extracellular signal-regulated protein kinase that stimulates neurogenesis and decreases the phosphorylation of the kinases JNK and apoptosis activators, and thus reduced Bax expression and increased expression of Bcl-2 antiapoptosis protein measured by western blot in ischemic brain lesions [54]. The reintroduction of O_2_ into ischemic tissue after reperfusion leads to the overproduction of reactive oxygen species and ONOO-, which increase the activity of the glutamate transporter and can overactivate N-methyl-D-aspartate (NMDA) receptors [55]. Thus, the antioxidant role of vitexin or even its inducing effect of constitutive antioxidant enzymes is paramount for the integrity of neural cells, especially since activation of the extra-synaptic NMDAR receptors contributes to excitotoxicity and cell death.

In this same model of induction of cerebral infarction induced by occlusion of the middle cerebral artery (MCAO) Jiang and contributors [56] observed that vitexin was also effective in decreasing levels of markers of oxidative stress, LDH, MDA, and NO; decreasing inflammation by attenuating the secretion of the proinflammatory cytokines IL-6 and THF-*α*, further reducing autophagy by increasing mTOR receptor expression and decreasing Beclin expression; suppressed and apoptosis by raising Bcl2 expression and reducing the expression of caspase-3 and Bax.

In a model of hypoxia-ischemia in mice C57BL/6 newborns, of both sexes, the pre-treatment with vitexin (30 and 60 mg/kg) significantly attenuated the volume of the infarct, protected the cells against atrophy and improved the neurofunctional recovery of these animals. In the same study, primary cultures of cortical neurons of C57BL/6 mice subjected to oxygen deprivation and glucose pre-treated with vitexin (10 *μ*mol/L) had increased cell viability, LDH levels and Ca^2+^ reduced Ca^2+^/calmodulin complex, which can activate the calcium/calmodulin II dependent protein kinase (CaMKII), involved in the activation of NF-*κ*B, was compromised. According to Min et al*.,*[57] the inhibition of phosphorylation of this pathway is the main pathway of neuroprotection conferred by vitexin, in addition to suppressing apoptosis by increasing the Bcl^−2^/Bax and caspase-3 protein ratio.

Pre-treatment of cortical neurons from C57Bl/6 E18 mice with vitexin (10 *μ*M) reduced the damage that excessive excitatory neurotransmitter glutamate and overactivation of N-methyl-D-aspartate (NMDA) receptors caused, as decreased viability cell death by apoptosis, high Bax/Bcl-2 ratio, as well as caspase-3 activation and intracellular Ca^2+^ elevation. Yang et al. [58] associated this effect with decreased expression of NR2B, an NMDA receptor subtype implicated in excitotoxicity, and reduction of calcium influx, which in excess would contribute to the increase in EO and to mitochondrial dysfunction observed in neurodegenerative diseases. In addition to this negative regulation of the glutaminic receptor, glutaminic acid, Chen et al. [59] (2016) also indicate the downregulation of the potential cation channel transient receptor V (TRPV1) by vitexin (10 or 100 *μ*M) as pathways of neuroprotective action, in addition to a significant reduction of cytokine levels (BACE-1), caspase-3, and cytosolic calcium levels in PC-12 human pheochromocytoma cells induced by the anesthetic isoflurane, and improved the concentration of the GSH and SOD antioxidants.

Activation of NMDAR receptors containing GluN2B by accumulation of A*β* may occur in the early stages of AD and cause imbalance in calcium homeostasis [60]. However, activation of postsynaptic NMDARs initiates plasticity and stimulates cellular survival and memory, and this feat is harnessed in the form of pharmacological treatment in AD by the drug memantine [55]. In Parkinson's hyperactivation of glutamine receptors is implicated in the development and maintenance of levodopa-induced side effects and the blockade of which can be used in the modulation of disabling dyskinesias [61].

In convulsions induced for pilocarpine in adult rats, vitexin (10 mg/kg) increased the latency period and reduced the frequency of seizures, thus increasing neural cell survival, and also increased the antioxidant enzymes SOD, catalase and GSH in the brain reduced by pilocarpine, and provided a significant reduction in the high NO and TBARS content. In this experiment ASEERVATHAM et al. [62] have observed that vitexin reduces excitotoxicity by negatively regulating the expression of mGluR1 and mGlu5 transcription genes of the NMDA receptor. Another important finding in this experiment and that can be targeted for future research for the benefit of PD is the reduction of MAO levels, which were increased after seizures induced by pilocarpine.

**Figure 2:**
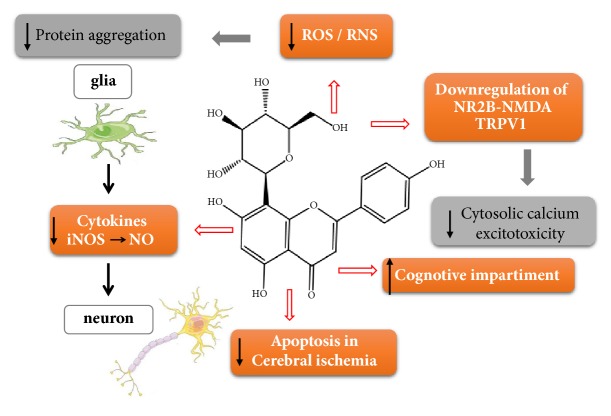
The major targets are involved in neurodegeneration and CNS injury that can be modulated by vitexin. The aggregation of toxic oligomers generates free radicals and exacerbates oxidative and nitrosative stress, in turn active as eminent and mortal signaling pathways, a self-perpetuating vicious cycle. Vitexin prevents an apoptoxic neuronal attack in acute brain lesions revealed by the increase of Bcl-2, and increases Bax and caspases-3 and attenuates a neurotoxicity induced by the release of calcium in NMDA receptors.

Although autophagy is required for the degradation of poorly folded and aggregated proteins that contribute to the toxicity observed in Parkinson's and Alzheimer's, in ischemia this mechanism is essential to reserve energy and provide metabolic substrates for survival; however its sustained activity and uncontrolled can digest vital amounts of cellular components and survival factors, leading to energy depletion, DNA fragmentation, and activation of cell death signaling pathways [58, 63]. From these results we also observed that similarly to other flavonoids, vitexin conferred neuroprotection by suppressing the gene expression of apoptotic signaling enzymes and the inflammatory signaling pathways activated by NF-kB. [14]. Modulation of these pathways also implicated in neurodegeneration may provide a potential treatment strategy for PD and AD by vitexin. These results show that vitexin negatively regulates the presynaptic NMDA expression and reduces the level of intracellular calcium exerts a neuroprotective effect, besides reducing the deleterious effects caused by acute injuries to the Central Nervous System (CNS) and the neurotoxic effects in the models experimental studies of Alzheimer's and Parkinson's induction, as summarized in Figure 2.

## 4. Conclusions

In the few studies that explore the effects of vitexin on the mechanisms implicated in the pathogenesis of neurodegeneration, it has demonstrated a great therapeutic potential to be explored in the reduction of oxidative stress, neuroinflammation, besides reducing the toxicity induced by the protein aggregates by inhibiting its aggregation or even purifying the toxic oligomers observed mainly in AD and PD. Recent reports deal more with the protective effects that vitexin plays against the injuries produced by hypoxia-ischemia, stroke, and the use of anesthetics widely used in the clinic. As flavonoid and by the observed effects vitexin has the ability to effectively cross the blood brain barrier and can act on a multiplicity of molecular targets, either by reducing the EO through its antioxidant effects or by even inducing the expression of the endogenous antioxidant enzymes.

The Vitexin further contributes to the reduction of neural cell death observed in neurodegenerative diseases and acute insults, through downregulation of proinflammatory and apoptotic signaling pathways and supraregulating cell survival pathways. Allied to these characteristics, vitexin proved to be safe and increased cell viability, improved cognitive and locomotor activities in in vivo models, suggesting a huge potential to be explored in the pharmacological therapy of neurodegenerative diseases, both in the cellular and underlying molecular events degeneration, and contributed to improving the disabling symptoms observed in AD and PD patients. Although this review and other recent studies demonstrate its therapeutic effects, further research is needed to substantiate existing findings and accelerate knowledge in this area.
